# Initial Method for Characterization of Tympanic Membrane Drug Permeability in Human Temporal Bones *In Situ*

**DOI:** 10.3389/fneur.2021.580392

**Published:** 2021-02-23

**Authors:** Samuel Early, Rong Yang, Xiyu Li, Zipei Zhang, Jens C. van der Valk, Xiaojie Ma, Daniel S. Kohane, Konstantina M. Stankovic

**Affiliations:** ^1^Eaton-Peabody Laboratories, Department of Otolaryngology – Head and Neck Surgery, Massachusetts Eye and Ear, Boston, MA, United States; ^2^Department of Otolaryngology – Head and Neck Surgery, Harvard Medical School, Boston, MA, United States; ^3^School of Medicine, University of California, San Diego, San Diego, CA, United States; ^4^Laboratory for Biomaterials and Drug Delivery, Department of Anesthesiology, Division of Critical Care Medicine, Children's Hospital Boston, Harvard Medical School, Boston, MA, United States; ^5^Robert Frederick Smith School of Chemical and Biomolecular Engineering, Cornell University, Ithaca, NY, United States; ^6^Leiden University Medical Center, Leiden, Netherlands; ^7^Department of Otolaryngology, Qilu Hospital of Shandong University, Jinan, China; ^8^Program in Speech and Hearing Bioscience and Technology, Harvard Medical School, Boston, MA, United States; ^9^Harvard Program in Therapeutic Science, Harvard Medical School, Boston, MA, United States

**Keywords:** otitis media, antibiotic, permeability, temporal bone, permeability enhancers, tympanic membrane

## Abstract

**Background and Introduction:** Acute otitis media is the most common reason for a visit to the pediatrician, often requiring systemic administration of oral antibiotics. Local drug therapy applied to the middle ear could avoid side effects associated with systemic antibiotic administration, however in the majority of patients this would require drugs to diffuse across an intact tympanic membrane. Experimental methods for testing trans-tympanic drug flux in human tissues *in situ* would be highly valuable to guide drug therapy development for local drug delivery to the middle ear.

**Materials and Methods:** A total of 30 cadaveric human temporal bones were characterized by trans-tympanic impedance testing to determine how steps in tissue processing and storage might impact intactness of the tympanic membrane and thus suitability for use in studies of trans-tympanic drug flux. Ciprofloxacin drug solutions of varying concentrations were then applied to the lateral surface of the tympanic membrane in eight samples, and middle ear aspirate was collected over the following 48 h to evaluate trans-tympanic flux to the middle ear.

**Results:** Tissue processing steps that involved extensive tissue manipulation were consistently associated with evidence of microperforations in the tympanic membrane tissue. Maintaining the tympanic membrane *in situ* within the temporal bone, while using an otologic drill to obtain transmastoid access to the middle ear, was demonstrated as a reliable, non-damaging technique for accessing both lateral and medial surfaces for trans-tympanic flux testing. Results in these bones demonstrated trans-tympanic flux of ciprofloxacin when administered at sufficiently high concentration.

**Discussion and Conclusion:** The study describes key techniques and best practices, as well as pitfalls to avoid, in the development of a model for studying trans-tympanic drug flux in human temporal bones *in situ*. This model can be a valuable research tool in advancing progress toward eventual clinical studies for trans-tympanic drug delivery to the middle ear.

## Introduction

Acute otitis media (AOM) is a common infectious disease in children, and the most common reason for a visit to the pediatrician (1, 2). Complications of untreated disease can include disease recurrence, hearing loss, progression to chronic otitis media, cholesteatoma, and meningitis; these complications and associated risks can persist into adulthood (3–6). Current clinical guidelines recommend exclusively systemic therapy when antibiotics are indicated, since locally applied antibiotic drops have only ever been shown to be effective at eradicating infection with presence of either tympanic membrane perforation or tympanostomy tube (5, 7, 8). In children with persistent serous effusion of the middle ear following AOM, placement of transtympanic ear tubes can aid topical antibiotics to reach the middle ear space, however for the majority of patients this is not a practical solution to avoid systemic antibiotic therapy, given the need for tubes to be regularly replaced and the general anesthesia requirement for this procedure in children (9, 10).

Prior studies have shown the ability of combined hydrogel and chemical permeation enhancers to promote antibiotic flux across an intact tympanic membrane, demonstrating complete elimination of non-typeable *Haemophilus influenzae* and *Streptococcus pneumoniae* infections in a chinchilla model *in vivo* without use of systemic antibiotic therapy (11–13). Achieving similar results in humans, however, is anticipated to be more challenging, given the greater thickness of the human tympanic membrane, larger volume of the middle ear space in humans, increased complexity of the human middle ear anatomy compared to the simple bulla in the rodent model, and current lack of preclinical data concerning tissue characteristics of human tympanic membranes as related to impedance and trans-tympanic drug flux. The current burden of adverse effects with systemic antibiotic therapy, however, nonetheless warrants further investigation to support development of non-systemic routes of administration, and a functional method of measuring trans-tympanic drug flux in human tissues *in situ* would support this development (3).

Numerous challenges are anticipated in the development of a human tissue *in situ* model for testing of trans-tympanic drug delivery. For one, although the effects of chemical permeation enhancers on diffusion properties of human skin have been extensively tested in prior research, relatively little is known about trans-tympanic diffusion properties of the human tympanic membrane (14). Furthermore, availability of fresh tissues is much more challenging in human vs. animal studies, given the reliance on cadaveric specimens and potential for increased tissue degradation due to post-mortem time delays and processing techniques. Addressing these limitations will be necessary in order to create robust and reliable models for testing trans-tympanic diffusion of novel drug formulations in human tissue *in situ*.

The current study describes key characteristics of fresh human cadaveric temporal bone samples, important considerations for measuring trans-tympanic membrane drug flux in human temporal bones *in situ*, and high-yield learnings from the process of developing and refining the diffusion testing model.

## Methods

**Figure 1:**
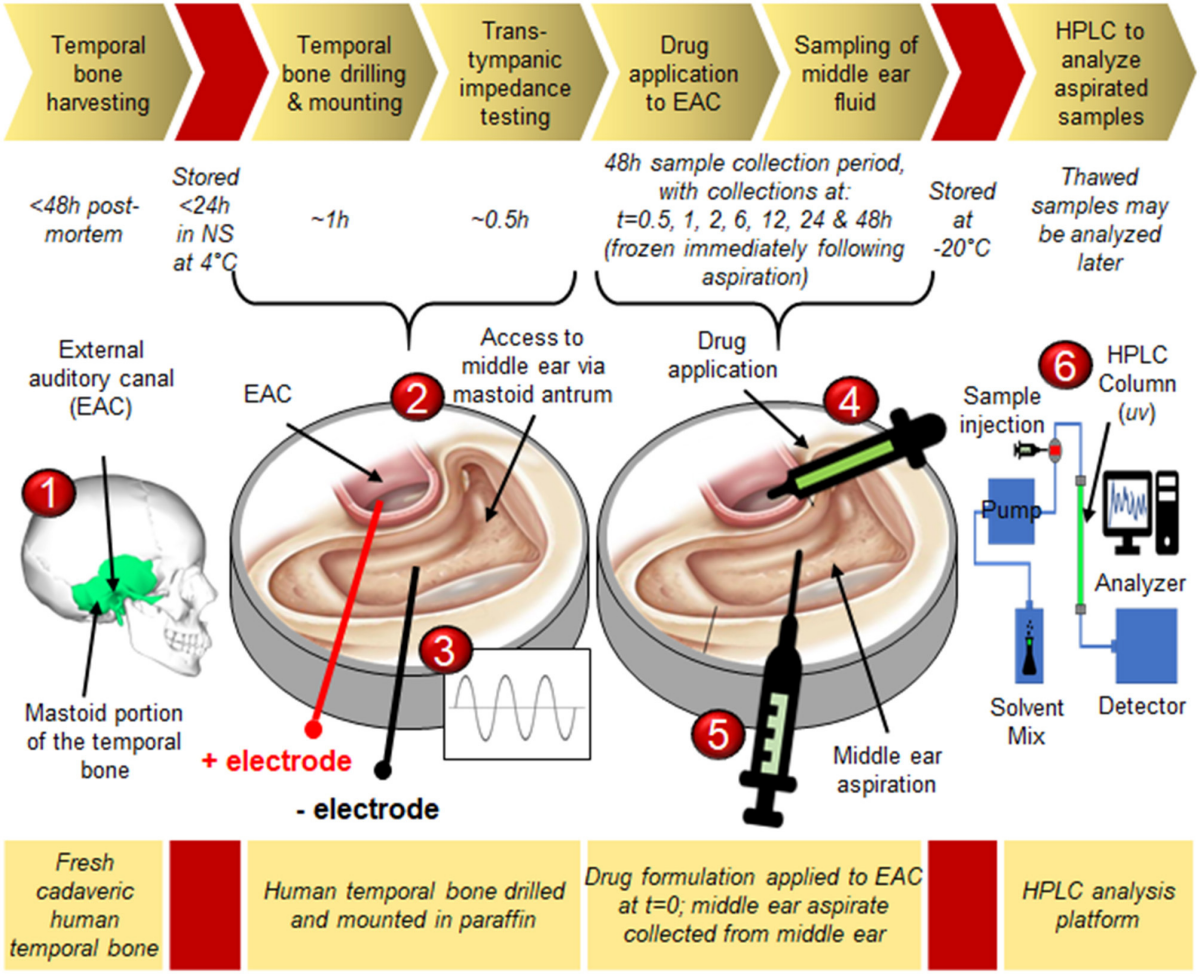
Experimental timeline for measurement of trans-tympanic drug delivery *in situ*: **1)** Cadaveric temporal bones are collected from the Mass Eye and Ear otopathology service; **2)** The mastoid portion of the temporal bone is carefully drilled away under a microscope to expose the middle ear space via the antrum and facial recess, and the drilled bone is mounted in paraffin to provide access to both the external auditory canal (EAC) as well as the middle ear space; **3)** Impedance testing confirms intactness of the tympanic membrane; **4)** The middle ear is loaded with phosphate-buffered saline (PBS), while drug is applied to the lateral surface of the tympanic membrane via the EAC; **5)** At defined time points the middle ear fluid is aspirated and replaced with fresh PBS; **6)** Aspirated samples are analyzed via high performance liquid chromatography to determine drug concentration with high degree of precision. Graphical representation of temporal bone seen in steps 2–5 is adapted from Jackler (15) (c) Chris Gralapp 2020.

Figure 1 shows the overall timeline for human temporal bone collection, processing and testing during the study (15).

### Temporal Bone Harvesting

Thirty human temporal bones were collected at autopsy at the Massachusetts General Hospital in Boston, MA. All patients were deceased from non-otologic causes, did not have any known history of prior otologic disease, and were fully de-identified. Collection of all specimens from cadaveric donors was completed within 48 h post-mortem. In 24 cases, temporal bones were provided from the autopsy service in normal saline at 4°C, where they had been stored for a maximum of 24 h since collection. In six cases the temporal bones had previously been frozen at −20°C for long-term storage. All frozen samples were thawed in normal saline at 4°C before use in the study. Of the six previously-frozen bones, two had undergone only a single freeze-thaw cycle before use in the study, while the remaining four had each undergone at least three freeze-thaw cycles.

### Temporal Bone Drilling and Mounting

**Figure 2:**
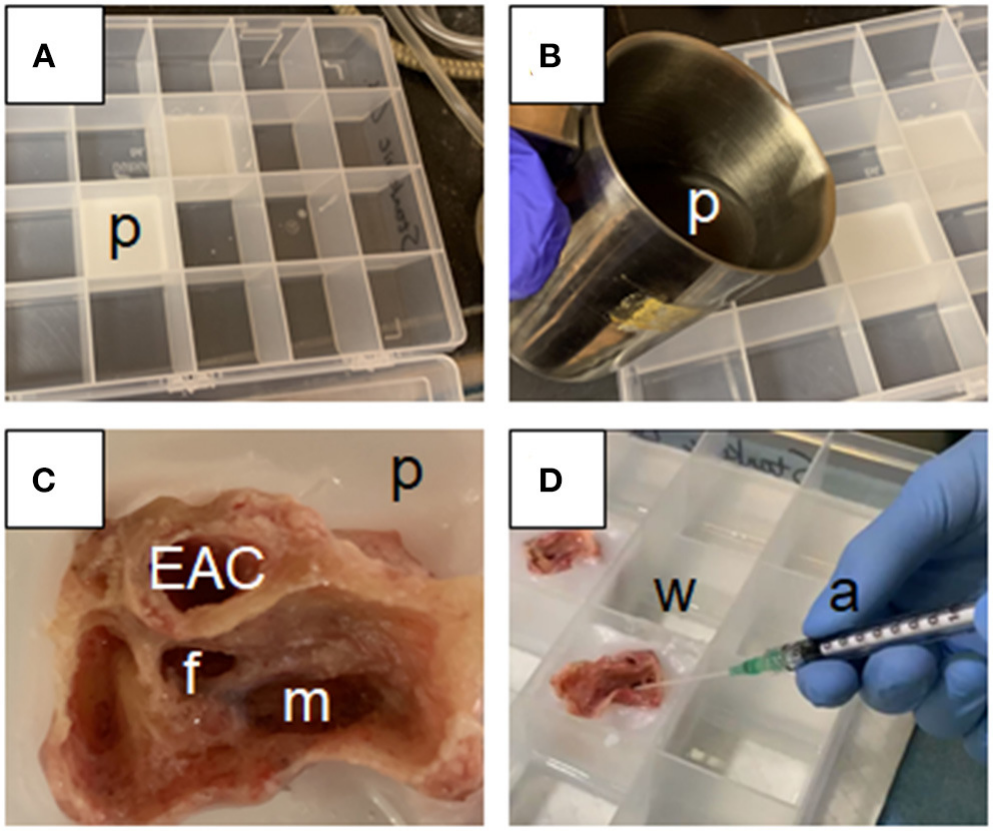
Experimental set-up for measurement of trans-tympanic drug delivery *in situ*: **(A)** Paraffin is heated to 60°C, poured into a clear plastic compartment organizer, and allowed to solidify to form a 1 cm deep paraffin base (p); **(B)** Once the base is solidified, additional hot paraffin (p) is added to an additional 1 cm depth; **(C)** The post-mastoidectomy temporal bone is set into the hot paraffin such that the external auditory canal (EAC) is oriented vertically, and becomes fixed in place as the cooling paraffin (p) forms a watertight shell surrounding the drilled mastoid (m), with access to the lateral surface of the tympanic membrane obtained via the EAC and to the medial surface via the facial recess (f); **(D)** Extra compartments within the compartment organizer are filled with water at 37°C to maintain humidity and consistent temperature throughout the experiment (w). A series of angiocatheters (a) are used to perform the following tasks throughout the experiment: filling of the middle ear space with normal saline via the antrum, applying drug formulation to the tympanic membrane via the EAC, and collecting middle ear aspirate via the facial recess.

In each temporal bone, an otologic drill was used to access the middle ear space via transmastoid facial recess approach (15). The external auditory canal (EAC) was inspected for patency, and any loose debris or cerumen was gently removed via suction. Particular care was taken at all times not to manipulate the skin of the EAC, any component of the ossicular chain, or the tympanic membrane itself. A temporal bone tray was prepared as shown in Figure 2A, where individual compartments in the tray were first filled 1/3 full with liquid paraffin, which was allowed to cool and form a solid base. Once the drilled temporal bones were ready, then additional liquid paraffin at 60°C was added to bring the compartment level up to 2/3 full as shown in Figure 2B. Drilled temporal bones were placed into the still-hot paraffin with the EAC in a vertical orientation, to as great a depth as possible without allowing spillover from the liquid paraffin into either the exposed EAC or middle ear space, as shown in Figure 2C. Paraffin was allowed to solidify at ambient temperature of 20°C while next steps were being completed.

Extraction of the tympanic membranes entirely from the surrounding temporal bone for placement in a diffusion cell apparatus was considered, however not implemented given the difficulties encountered in extracting the tympanic membrane intact and without causing microperforations in the process; similar difficulties have limited prior studies in animal models to use of the entire bulla rather than an extracted tympanic membrane (12). Even minor manipulation of the ossicular chain was found to be associated with increased incidence of microperforation, as well as reduced baseline trans-tympanic impedance measurements, and for these reasons flux testing was only carried out in temporal bones *in situ* rather than in extracted tympanic membranes.

### Trans-tympanic Impedance Testing

The middle ear was filled with phosphate-buffered saline (PBS) via the mastoid antrum at volume of 1.2–2.0ml depending on individual temporal bone capacity, and the lateral surface of the tympanic membrane was visually inspected using binocular microscopy for any evidence of trans-tympanic leakage from the middle ear space. Tympanic membranes with evidence of frank perforation by this method were removed from consideration for the remainder of the study, and replaced in the sample with a fresh cadaveric specimen. The EAC was then also filled with phosphate-buffered saline, and electrodes placed into both the middle ear space and EAC to measure trans-tympanic electrical impedance using HP Hewlett Packard 33120A 15 MHz Function/Arbitrary Waveform Generator and EXTECH Instruments Industrial MultiMeter EX510 as described previously, with the exception that no lower cutoff was used to presume intactness vs. non-intactness of the tympanic membranes (13, 16). Saline was then gently aspirated from the EAC, taking care not to manipulate either the canal wall skin or the tympanic membrane itself. Unfilled compartments in the temporal bone tray were filled to midlevel with water at 37°C to maintain ambient humidity, and the temporal bone tray was then placed in an incubator at 37°C; temporal bones remained in the incubator for the duration of the study.

### Drug Application to External Auditory Canal

Two drug solutions were used in this study. The first solution, 1% w/v ciprofloxacin in normal saline, is equivalent in concentration to prior studies and to clinically available ear drops (13). The second solution, 4% w/v ciprofloxacin in normal saline, represents the highest stable concentration of ciprofloxacin that could be achieved in our lab without notable precipitation after 48 h at 37°C. A 300 μl volume of drug solution was applied via syringe to the EAC and lateral surface of the tympanic membrane in each temporal bone. Among the 24 temporal bones that had never been previously frozen, three bones received 1% ciprofloxacin solution, while five bones received 4% ciprofloxacin solution. The remaining 16 fresh-never-frozen temporal bones were repurposed for a separate study. Of the six previously-frozen temporal bones, three bones received 1% ciprofloxacin solution, while three bones received 4% ciprofloxacin solution.

### Sampling of Middle Ear Fluid

At 30 min following drug application, a syringe was placed medial to the tympanic membrane via facial recess approach, and solution from the middle ear space was aspirated as shown in Figure 2D. The first milliliter of aspirated volume was considered to be the “A” sample and to be most representative of trans-tympanic drug flux. Following collection of the “A” sample, any remaining volume from the middle ear space or any fluid which may have pooled externally to the temporal bone was then aspirated as the “B” sample. Primary compositional analysis for the study was intended to focus on results of the “A” sample, while collection of the “B” sample was intended as a quality control in case any of the drug solution were to enter the middle ear space by spillage from the EAC, rather than through trans-tympanic flux. Following aspiration of these samples, the middle ear space was then refilled with fresh PBS. Repeat samplings from the middle ear space using the same protocol were repeated at 1, 2, 6, 12, 24, and 48 h following time of initial drug application. Each sample was stored immediately after collection at −20°C.

### High Performance Liquid Chromatography (HPLC) to Analyze Aspirated Samples

Samples were analyzed using high-performance liquid chromatography to assess ciprofloxacin concentration in middle ear aspirate as previously described (12). Comparison was made at all time points between “A” and “B” sample concentrations, and in any cases where “B” sample values exceeded those of “A” samples, it was presumed that passage of drug into the middle ear space was likely due a route other than trans-tympanic. In these cases, samples were discarded from consideration in the study, and replaced in the study population by a new cadaveric specimen.

### Tracking of Key Operational Parameters for Reliable and Reproducible Results

In developing a novel technique for testing trans-tympanic membrane drug flux, care was taken to record all potential variables related to tissue processing steps and processing times, not only from a technical but also operational perspective. Standard practice for storing human temporal bones at our institution, for example, has previously involved wrapping the bones tightly in plastic and freezing at −20°C until a convenient time of use, and these methods had proven sufficient in the past to support research findings related to mechanical properties of key middle and inner ear structures (17–19). We did not take for granted that previously-frozen tissues would produce reliable results, however, and thus sought to include both previously-frozen and fresh-never-frozen tissue samples for purpose of comparison. All processing steps from time of donor death until completion of flux testing were recorded meticulously and evaluated for potential effect on data quality and reliability. Pitfalls and challenges related to mounting tissues in paraffin, drug application to the EAC, sampling of fluid from the middle ear space, maintaining tissue quality over time and preventing spillage were all recorded and critiqued throughout the development of the protocol.

## Results

### Trans-tympanic Impedance Measurements

**Figure 3:**
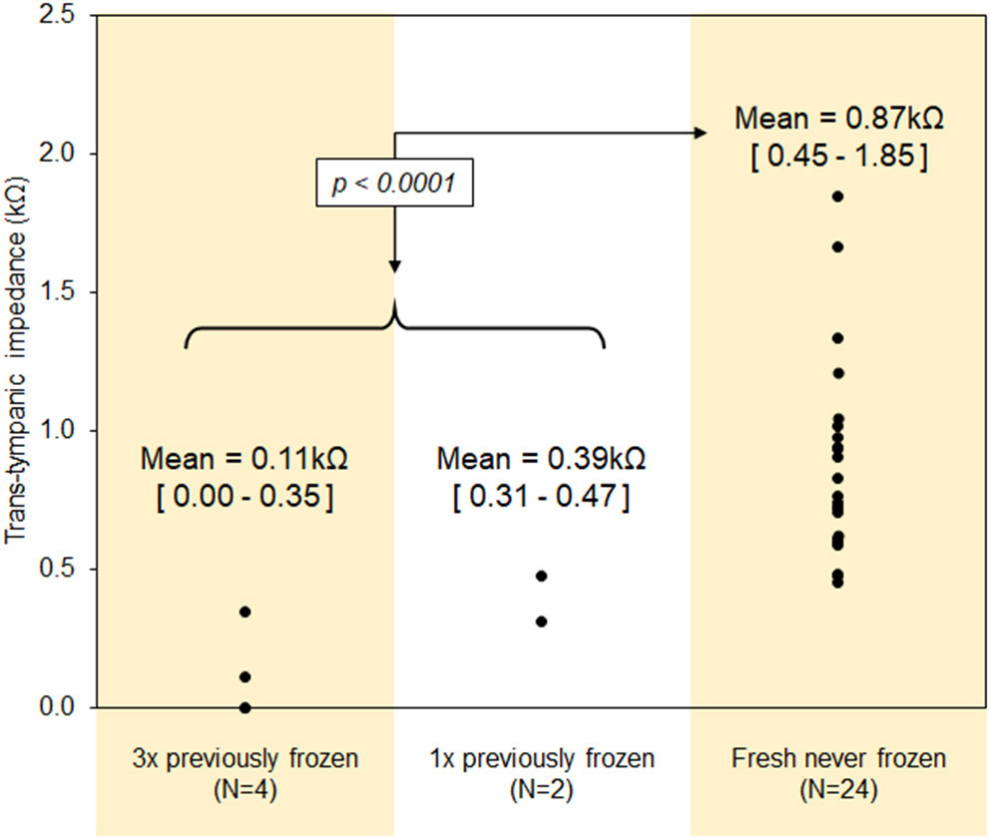
Trans-tympanic impedance characteristics of the human tympanic membrane.

Figure 3 demonstrates trans-tympanic impedance characteristics of the 30 human temporal bones evaluated in this study. Trans-tympanic impedance for fresh-never-frozen temporal bones was significantly higher than for previously-frozen bones; sample sizes were not large enough to determine relative effect of single vs. multiple freeze-thaw cycles. Mean trans-tympanic impedance in fresh-never-frozen temporal bones was 0.86 kΩ, ranging from a minimum of 0.45 kΩ to maximum of 1.85 kΩ. Tympanic membranes for all 30 bones were intact by visual inspection, however later flux testing in all six previously-frozen bones demonstrated a very large trans-tympanic drug flux, with middle ear drug concentration spiking to >50 μg/ml as early as 30 min after placement of drug solution into the EAC. This was interpreted as evidence of likely preexisting microperforations in these tympanic membranes, and as such, any temporal bone with baseline impedance less than or equal to the upper limit for previously-frozen temporal bones (0.47 kΩ) were excluded from further analysis in this study.

### Temporal Bone Processing

Processing times for human temporal bones used in this study are described in Table 1. Among the 24 fresh-never-frozen temporal bones, the mean time post-mortem until placement in normal saline at 4°C was 28 h (range 12–48 h), and the mean time from placement in normal saline until start of flux testing was 15 h (range 4–20 h). Mean time post-mortem until start of flux testing was thus 43 h, however with a large range from 32 to 63 h. The time delay from post-mortem until extraction and placement in saline was primarily a function of established protocols within the otopathology lab at our institution, while delay from tissue collection until start of testing were primarily related to researcher availability at time of receiving notification from the otopathology service.

**Table 1:**
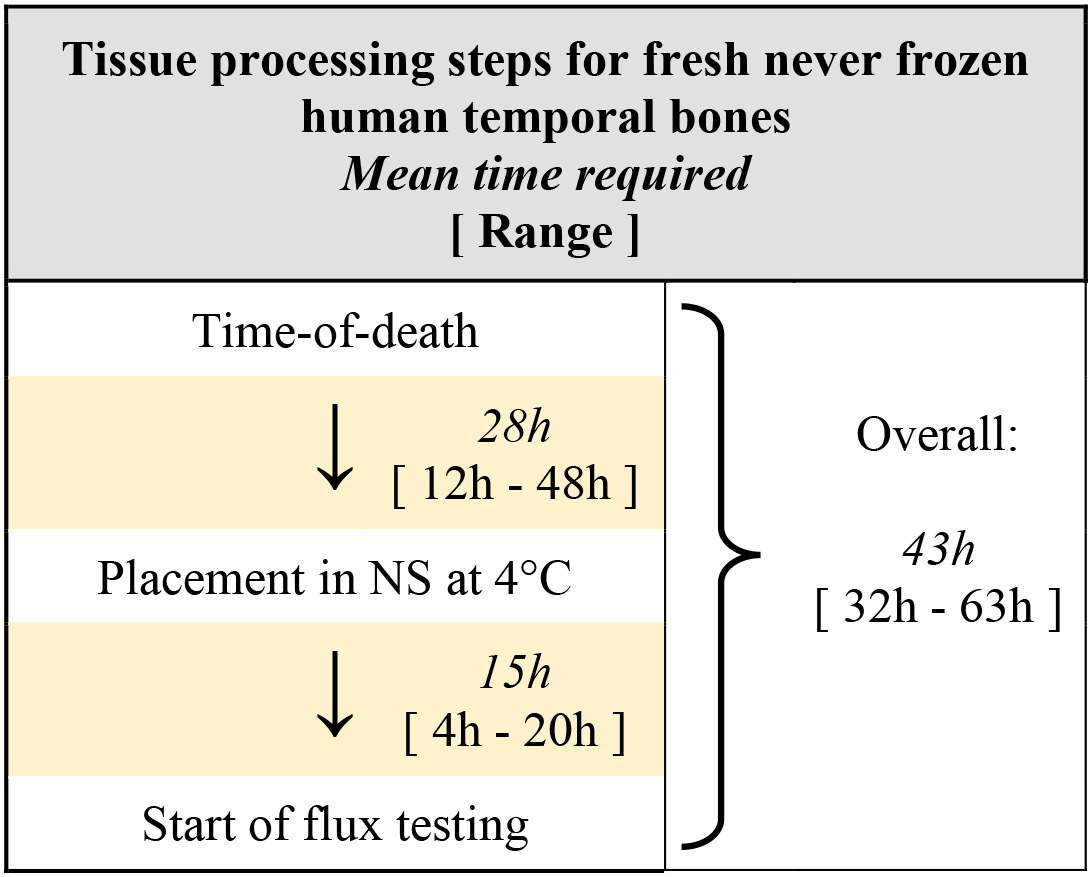
Timing for tissue processing steps, from time-of-death to start of flux testing.

### Relationship Between Processing Time and Trans-tympanic Impedance

**Figure 4:**
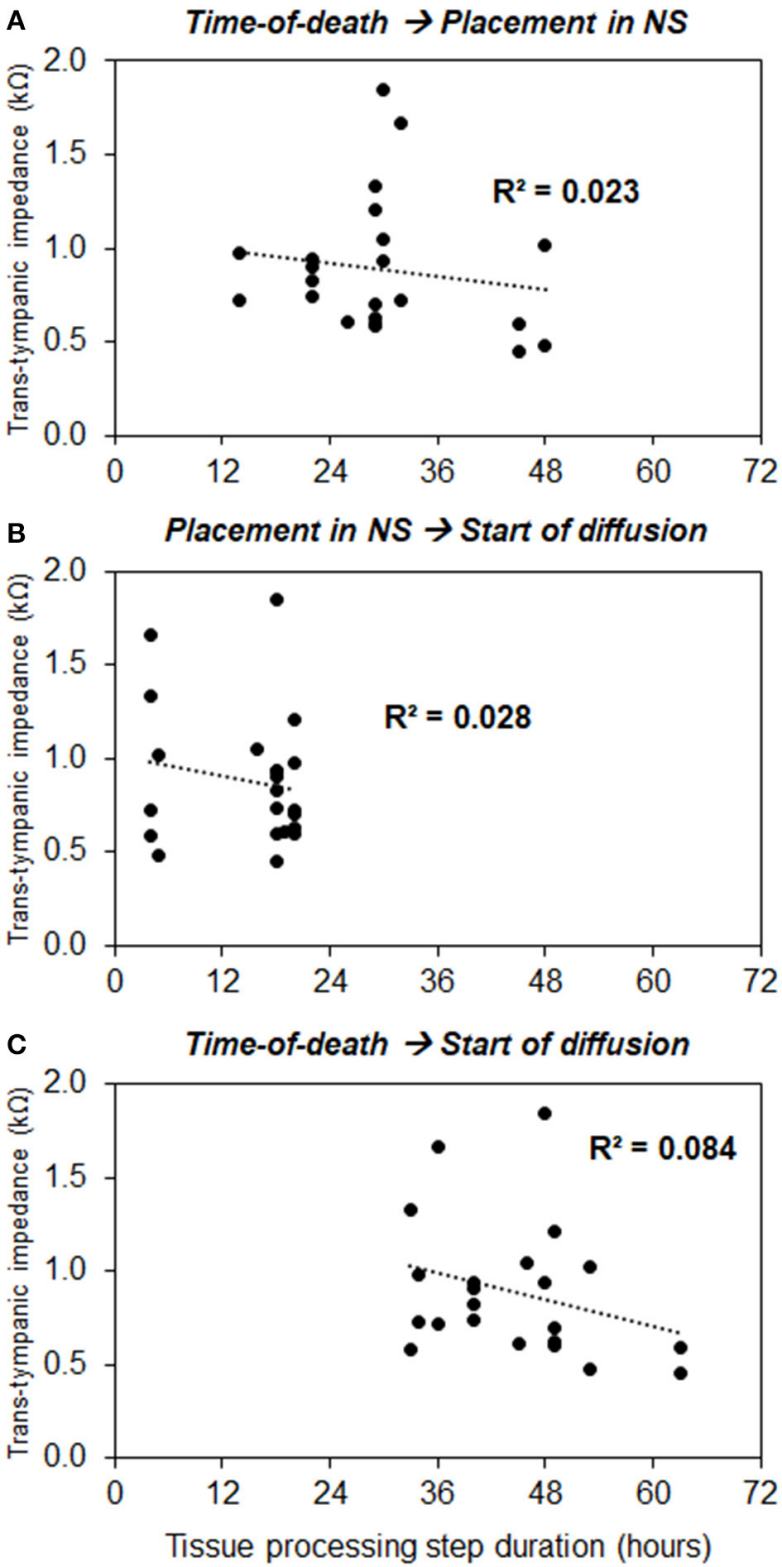
Correlation between processing times and baseline trans-tympanic impedance measurements, with processing time measured as follows: **(A)** From time-of-death to placement in normal saline, **(B)** From placement in normal saline to start of diffusion, and **(C)** From time-of-death to start of diffusion.

No significant correlations were found between any time requirements for processing of temporal bones and resulting trans-tympanic impedance, as shown in Figure 4. The lack of a convincing trend was the same regardless of whether only time delay post-mortem to storage in saline at 4°C was considered (Figure 4A), or only time in saline at 4°C before flux testing was considered (Figure 4B), or whether total processing time post-mortem until start of flux testing was considered (Figure 4C). Although each time parameter was negatively correlated with trans-tympanic impedance, in all cases *R*^2^ was <0.1.

### Relationship Between Trans-tympanic Impedance and Drug Flux

As noted previously under “Trans-tympanic impedance measurements,” when drug solution was placed into the EAC of previously-frozen temporal bones, the result was an immediate spike in drug flux. Middle ear drug concentrations reached >50 μg/ml at 30 min after drug placement in the EAC, establishing that even the upper limit of trans-tympanic impedance measured in previously-frozen temporal bones, 0.47 kΩ, was not representative of an intact tympanic membrane. All previously-frozen temporal bones were therefore excluded from further analysis.

**Figure 5:**
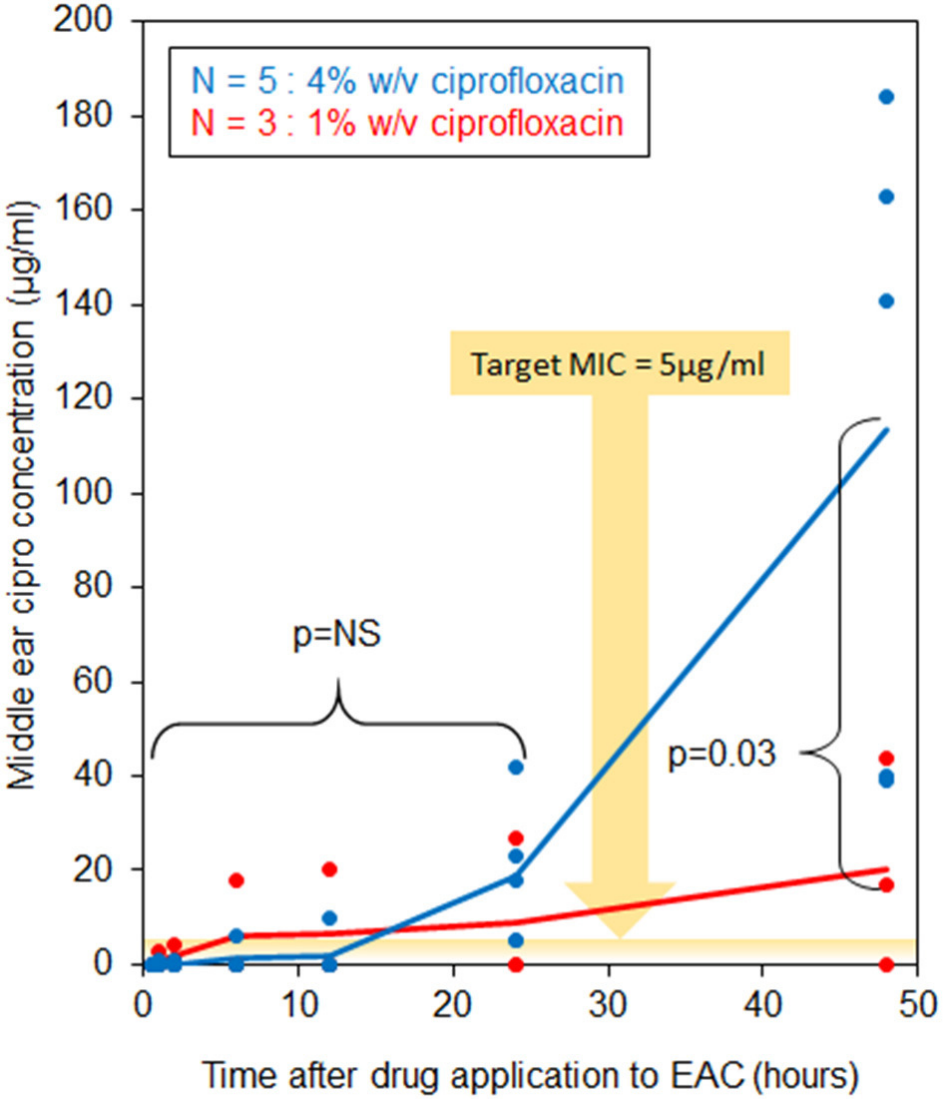
Middle ear ciprofloxacin concentration over time.

**Figure 6:**
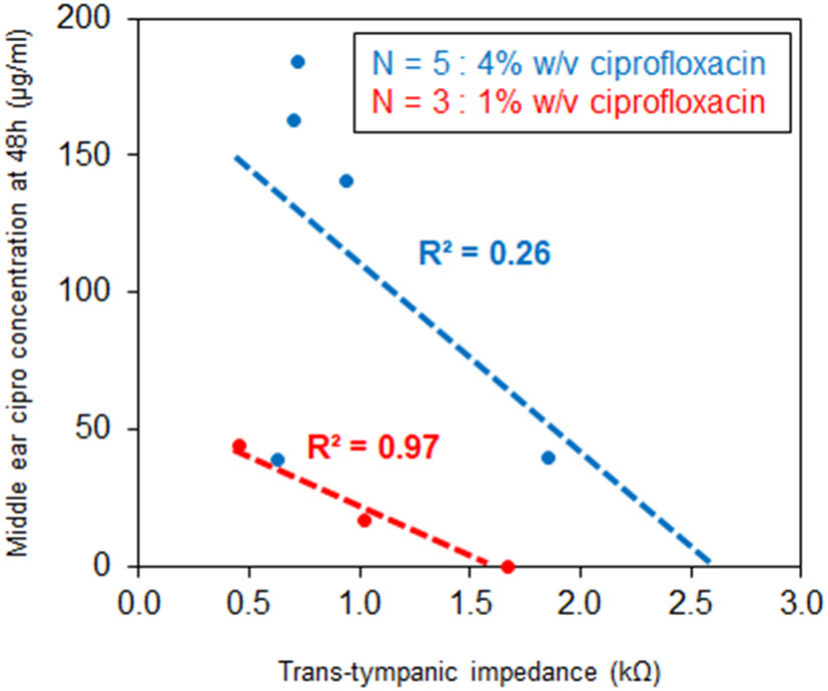
Correlation between trans-tympanic impedance and middle ear ciprofloxacin concentration at 48 h.

At 48 h after drug application in fresh-never-frozen specimens, temporal bones with 4% w/v ciprofloxacin applied to the lateral surface of the tympanic membrane demonstrated significantly higher trans-tympanic drug flux than did temporal bones with application of only 1% ciprofloxacin (*p* = 0.03) as shown in Figure 5. No significant drug flux was noted with either drug formulation at any time point of 2 h or less, and no significant difference between the two formulations for any time points at 24 h or less. Negative correlations were seen between baseline trans-tympanic impedance and middle ear drug concentration at 48 h, as seen in Figure 6. Greater variability in middle ear drug concentration was seen in temporal bones with both 4% w/v ciprofloxacin and lower baseline trans-tympanic impedance, however, limiting the strength of correlation between impedance and drug flux in this group. Similar although weaker trends were seen at the 24 h time point as well.

### Key Operational Parameters for Reliable and Reproducible Results

The following findings represent key insights from the process of developing the trans-tympanic flux model, including guidance on avoiding some potential pitfalls, and are intended to aid other researchers in the further advancement of these techniques:

1. *Proper positioning and orientation of temporal bones within paraffin base is critical to prevent leaks*. Vertical orientation of the EAC was critical to prevent drug solution from exiting the EAC by a non-trans-tympanic route, and testing of separate “A” and “B” samples as described in the methods allowed for high degree of confidence that drug could only be entering the middle ear space via trans-tympanic flux.
2. *Maintenance of humidity and temperature is critical to preventing early tissue degradation*. A tight-fitting lid on the temporal bone tray helps to prevent contamination, control odor and maintain humidity throughout the duration of the experiment. Lack of humidity control initially led to several operational problems, including tissue desiccation as well as increased fluid absorption from the middle ear space by residual soft tissues and associated difficulty in collecting adequate sample volumes for analysis.
3. *The ideal setting for this type of research is a high-volume hospital with rapid access to new cadaveric specimens and a dedicated team for collection and storage of temporal bone tissues*. Regular availability of and communication with the pathology service at our facility was critical to support timely tissue acquisition. The findings of this study demonstrated significantly reduced trans-tympanic impedance of previously-frozen tissues compared to use of fresh-never-frozen tissues, and furthermore showed that in previously-frozen temporal bones detection of significant flux within the middle ear space occurred almost immediately after drug application to the EAC. This finding was highly suspicious for the presence of tissue microperforations as a result of the freezing process; as regards baseline tympanic membrane integrity, best results were achieved by using only fresh-never-frozen tissues. Additionally, we found that ready availability of research team members with the requisite expertise for precise and rapid drilling of human temporal bones is necessary in order to minimize time delays from tissue availability to start of flux testing.

## Discussion

To our knowledge, this study represents the first attempt to develop a model for characterizing trans-tympanic drug flux in human cadaveric tissues *in situ*, including first attempt to characterize trans-tympanic impedance and relationship to steps in tissue collection and processing. As such, it is an important step in developing the techniques necessary to support research into trans-tympanic drug delivery, and to continue innovation in this field.

### Baseline Trans-tympanic Impedance Characteristics

The results of this study support the concept that baseline trans-tympanic impedance can be a valuable predictor of tympanic membrane intactness, and thus a non-destructive method to assess sample quality for studies of trans-tympanic drug diffusion *in situ*. This finding is supported not only by the clear association of high-risk tissue processing steps with both reduced baseline impedance and suspiciously high drug flux at early time points (Figure 3), but also by the association between tympanic membranes with higher baseline impedance showing consistently lower middle ear drug concentrations at time points up to 48 h (Figure 6). We note, however, that baseline trans-tympanic membrane impedance values obtained in this study are significantly lower than comparisons from prior literature using either *in vitro* human skin or *in situ* animal models (12, 14, 20). Several factors could have accounted for this difference, including unique tissue properties of the human tympanic membrane, tissue degradation during time required for post-mortem processing steps, and complex anatomy of the human temporal bone compared to the isolated tympanic membrane.

Freezing temporal bones yielded consistent evidence of microperforations and should be avoided, limiting final analysis only to fresh tissues *in situ*. Every temporal bone still required a minimum 12 h from time of donor death until extraction, however, and a further minimum of 4 h in saline at 4°C before initiation of flux testing. Compared to prior studies with either human skin or animal tissues, the main difference in processing time is the post-mortem delay until tissues could be collected. Animal tissue studies have been able to appropriately time the animal sacrifice and proceed immediately to tissue harvesting, while studies with human skin have been able to immediately freeze living donor samples in liquid nitrogen at −70°C (12, 14). The current study's reliance on cadaveric specimens requires a longer time delay post-mortem until tissue collection, as well as several additional hours for otologic drilling and mounting samples in paraffin. If there were a notable effect of the processing time delay on baseline trans-tympanic impedance, then we would expect it to become more pronounced in association with longer initial delays, however such an effect is not seen in our data (Figure 4).

Complex anatomy of the human temporal bone may also have contributed to lower trans-tympanic impedance measurements, given that measurements *in situ* would not be limited to impedance only across the tympanic membrane, but also in parallel through the surrounding canal wall skin, connective tissues and temporal bone. Given the relatively high impedance even of intact skin, however, much less the underlying connective tissues and bone, the relative contribution of these impedance in parallel would not account for much variation in trans-tympanic measurements.

Beyond the previously noted considerations related to post-mortem processing time and complex temporal bone anatomy *in situ*, the primary remaining consideration for lower trans-tympanic impedance values in these specimens would be unique tissue properties of the human tympanic membrane itself, compared to either animal tympanic membranes or human skin tissues tested in prior research. Compared to prior research using full-thickness human skin samples, the human tympanic membrane does differ significantly in compositional layers, substituting a lamina propia and mucosal layer for the dermis; it is also much thinner (50–120 μm for the tympanic membrane vs. up to 1.6 mm for a full thickness skin graft), which could explain reduced impedance measurements across the human tympanic membrane compared to across human skin samples (21–23).

It is always possible that the post-mortem processing time required for this study is still too long to maintain viable intact tissues. Further characterization of tympanic membrane characteristics at time points even closer to time of donor death would be necessary to better assess the extent to which these divergent values for trans-tympanic impedance are consistent with tympanic membrane intactness, or if a different relationship is relevant for these samples. Such characterization will be necessary to determine to what extent fresh-never-frozen cadaveric temporal bone specimens can reliably serve to model trans-tympanic membrane drug flux in living tissues.

### Implications for the Study of Trans-tympanic Drug Diffusion

This study demonstrates ability of ciprofloxacin to cross the tympanic membrane in human temporal bones *in situ*, reliably achieving a minimum inhibitory concentration (MIC) >5 μg/ml in the middle ear space within 24 h of drug administration to the EAC, well above the target MIC expected to be effective against common middle ear pathogens (24–27). We note that this effect appears positively associated with ciprofloxacin concentration in solution, and negatively associated with baseline trans-tympanic impedance (Figures 5, 6). Flux measurements may have been enhanced by positioning of temporal bone samples, in that drug solution maintained constant contact with the tympanic membrane surface during the course of testing; the measured flux aligns well, however, with previous results in a chinchilla model *ex vivo* (16).

That some drug flux is seen, and that it is dependent on drug formulation, does support further investigation into trans-tympanic diffusion properties in human tissues *in situ*. In only one sample was zero flux observed, and this case was limited to use of the lower 1% w/v ciprofloxacin solution. In all cases in which the higher 4% solution was used, flux above the target MIC was seen no later than 24 h after drug application. As shown in prior *in vivo* studies, the potential exists for increased tympanic membrane permeability in the setting of acute inflammation and associated immune response, and with administration of diffusion-enhancing agents (13). As such, future *in vivo* studies in humans may demonstrate even greater levels of trans-tympanic drug flux than what this study has achieved.

## Conclusion

This study describes techniques that can be used to further develop investigations into trans-tympanic drug diffusion in human temporal bones, such as the delivery of antibiotic or other drug therapy to the middle ear without the need for systemic oral therapy. We have identified, characterized and attempted to address limitations that are specific to use of human tissues and which were not previously understood, such as the impact of tissue processing steps on tissue feasibility for studies of trans-tympanic diffusion. Mounting of human temporal bones in paraffin to form a custom-fitted, waterproof seal is a novel approach that can allow for trans-tympanic flux testing in a controlled and anatomically accurate manner, without the need to risk damage to the tympanic membrane through extraction of an isolated tissue sample. We emphasize that best results regarding tissue integrity are maintained with minimizing tissue processing time and number of steps, in particular given the unavoidable minimum 12 h time delay post-mortem before tissues are available for research use. This time delay represents the primary difference in tissue processing time compared to prior animal studies, and as such further investigations should attempt to minimize as much as possible this time delay in order to better validate its impact on baseline trans-tympanic impedance.

This paper outlines a novel, functional method of measuring trans-tympanic drug flux in human tissues *in situ*, key findings from which can support novel research on the topic of trans-tympanic drug diffusion. Future research can leverage key learnings and best practices from this study to avoid potential pitfalls when working with highly valuable and difficult-to-acquire fresh human tissue samples.

## Data Availability Statement

The raw data supporting the conclusions of this article will be made available by the authors, without undue reservation.

## Ethics Statement

Ethical review and approval was not required for the study on human participants in accordance with the local legislation and institutional requirements. Written informed consent for participation was not required for this study in accordance with the national legislation and the institutional requirements.

## Author Contributions

KS and DK jointly conceived of the project and supervised all the work. SE, RY, KS, and DK designed the study. SE, JV, and XM collected data from specimens, while chromatography analysis was performed by XL, RY, and ZZ. Primary analysis and interpretation was then performed by SE, with input from all authors. SE, KS, and DK wrote the manuscript. All authors edited the manuscript and approved the final version.

## Conflict of Interest

The authors declare that the research was conducted in the absence of any commercial or financial relationships that could be construed as a potential conflict of interest.
